# Seasonal evaluation and mapping of aboveground biomass in natural rangelands using Sentinel-1 and Sentinel-2 data

**DOI:** 10.1007/s10661-023-12133-5

**Published:** 2023-11-28

**Authors:** Monde Rapiya, Abel Ramoelo, Wayne Truter

**Affiliations:** 1 Department of Plant and Soil Sciences, University of Pretoria, Pretoria, 0001 South Africa; 2 Centre for Environmental Studies, Department of Geography, Geoinformatics and Meteorology, University of Pretoria, Pretoria, 0001 South Africa

**Keywords:** Rangeland, Aboveground biomass, Leaf area index, Sentinel-1, Sentinel-2

## Abstract

Rangelands play a vital role in developing countries’ biodiversity conservation and economic growth, since most people depend on rangelands for their livelihood. Aboveground-biomass (AGB) is an ecological indicator of the health and productivity of rangeland and provides an estimate of the amount of carbon stored in the vegetation. Thus, monitoring seasonal AGB is important for understanding and managing rangelands’ status and resilience. This study assesses the impact of seasonal dynamics and fire on biophysical parameters using Sentinel-1 (S1) and Sentinel-2 (S2) image data in the mesic rangeland of Limpopo, South Africa. Six sites were selected (3/area), with homogenous vegetation (10 plots/site of 30m^2^). The seasonal measurements of LAI and biomass were undertaken in the early summer (December 2020), winter (July–August 2021), and late summer (March 2022). Two regression approaches, random forest (RF) and stepwise multiple linear regression (SMLR), were used to estimate seasonal AGB. The results show a significant difference (*p* < 0.05) in AGB seasonal distribution and occurrence between the fire (ranging from 0.26 to 0.39 kg/m^2^) and non-fire areas (0.24–0.35 kg/m^2^). In addition, the seasonal predictive models derived from random forest regression (RF) are fit to predict disturbance and seasonal variations in mesic tropical rangelands. The S1 variables were excluded from all models due to high moisture content. Hence, this study analyzed the time series to evaluate the correlation between seasonal estimated and field AGB in mesic tropical rangelands. A significant correlation between backscattering, AGB and ecological parameters was observed. Therefore, using S1 and S2 data provides sufficient data to obtain the seasonal changes of biophysical parameters in mesic tropical rangelands after disturbance (fire) and enhanced assessments of critical phenology stages.

## Introduction

Tropical rangelands are the principal rangeland worldwide, which covers approximately 20% of the surface land, and half of these rangelands originated in the African continent. They occupy nearly two-thirds of the land and 50% of the Southern African Development Community region (SADC) (Ramoelo et al., 2018; Tsalyuk et al., 2017). Above one-third (35%) of the land in South Africa is occupied by tropical savanna rangelands and are grouped as either dry or wet (mesic) (Mucina et al., 2006; Ramoelo et al., 2018; Urban et al., 2021). They consist of grasslands and diverse woody plants as the major vegetation types. Several studies highlight the contribution of tropical savanna rangelands to the rural economy as one of the vital roles in developing countries, like South Africa (Ramoelo et al., 2012; Shackleton et al., 2002).

The existence of native forage grasses that play a vital role in extensive grazing worldwide makes these rangelands preferable for both communal and commercial farmers (Skidmore et al., 2010). Rangelands provide natural grazing as a significant resource for extensive production (livestock), one of the primary sources of income in the majority of rural areas of South Africa (Ramoelo et al., 2012; Shackleton et al., 2002). These savanna rangelands are also used to regulate agroecological functions like the provision of space for the cultivation of sources of feed for humans and livestock, such as grains (maise, sorghum, and forage legumes) (Melillo et al., 1993). They also profoundly influence the rural society’s biogeochemistry changes and environment (Rajpal, 2014). This long-term sustainability of the agricultural industry is an important issue in food security for the entire world (Vågsholm et al., 2020).

However, these rangelands are exposed to various factors caused by humans and the environment. Numerous reports have highlighted their vulnerability to degradation resulting from mismanagement interventions, including overgrazing, unsustainable agrarian practices, intense human exploitation, and inappropriate land use, which have been documented worldwide (He & Mui, 2010; Ramoelo et al., 2012; Skidmore et al., 2010). Furthermore, the increasing frequency of climate change events is regarded as one of the most substantial challenges of the twenty-first century (Intergovernmental Panel on Climate Change (IPCC), 2007, 2021.

Recent reports have indicated that if measures to eradicate vegetation are not successful, approximately 12 million hectares of rangelands worldwide could become unsuitable for grazing (Urban et al., 2021). These factors contribute to fluctuations in seasonal forage availability for animals, which negatively impact the economies of developing countries like South Africa, as they heavily rely on the rural economy derived from livestock production (James et al., 2007; Ramoelo et al., 2012; Shackleton et al., 2002). In fact, the disturbance in rangelands was estimated to result in an annual economic loss of approximately ZAR 760,000 (equivalent to $45,000 USD) per 10 km^2^ livestock farm (Avenant, 2015).

Addressing these challenges requires vegetation assessment and the availability of scientific data on rangeland conditions under different management interventions (Everson & Hatch, 1999; Ramoelo et al., 2012). Such information can help land users, and policymakers understand the dynamics of each rangeland, from local properties to international levels, and highlight the need for existing environmental management policies (Climate change 2021 report of the IPCC, 2021; Living Planet Report, 2020; IPBES, 2019). By prioritizing data-driven decision-making, stakeholders can gain insights into the state of rangelands and make informed choices to mitigate degradation and promote sustainable land management practices.

In this context, the development of sustainable tools to monitor environmental resources from property to the international level was also discussed as a significant issue that must be included in each country’s environmental policy and framework documents (integrated management of natural resources, land, and land degradation (IMNRLLD, 56). That was a supplementary framework protocol to the policies from UNCCD, CBD, and UNFCCC (NEPAD, 2003). Hence, two methods have been developed and used to monitor rangeland status and productivity since the early eighteenth century. The traditional field method is the most commonly employed tool for rangeland assessment due to its simplicity and generally reliable results (De Luca et al., 2022). Although this field’s traditional method cannot measure or predict rangeland trends and the future, it only tells what is available now. It is also time-consuming and susceptible to challenges posed by vast geographic areas, particularly in extensive rangeland ecosystems (De Luca et al., 2022; Huang et al., 2018; Lu et al., 2016; Sibanda et al., 2021; Urban et al., 2020).

On the other hand, remote sensing has the ability to overcome these constraints. Remote sensing has become a valuable tool for monitoring extensive rangelands, as it offers a unique angle and synoptic view for monitoring vegetation status and productivity that complements the field’s traditional vegetation data (Nuthammachot et al., 2022). Due to the advantages of remote sensing, it has a good and valuable history in vegetation assessment and monitoring worldwide. Several studies have effectively utilized remotely sensed data to monitor rangeland status and productivity, as evidenced by research conducted by Sun et al., (2019), Liu et al., (2017), Lu et al., (2016), and Mutanga & Skidmore, (2004). However, previous applications of remote sensing tools for vegetation assessment encountered certain limitations, including challenges posed by factors like cloud cover, saturation during peak productivity, and economic feasibility, as discussed in studies by Mutanga et al., (2012), Ramoelo et al., (2012), Piñeiro et al., (2006), and Mutanga & Skidmore, (2004). Clouds present difficulties for optical remote sensing tools like Sentinel-2 that rely on visible and near-infrared light, which can be obstructed by cloud cover (Mutanga & Skidmore, 2004).

Though, recent advancements in remote sensing technology, particularly the use of synthetic aperture radar (SAR), have mitigated some of these challenges. SAR sensors operate actively by emitting microwave signals, which can penetrate cloud cover and can provide all-weather capabilities for monitoring vegetation. The availability of free high-temporal and high-spatial resolution remote sensing data, such as those from the Copernicus Sentinel mission, has significantly improved the accuracy of vegetation monitoring and mapping, making it a valuable tool for rangeland assessment even in the presence of cloud cover. These challenges are mitigated through the utilization of structural components, such as red-edge-based vegetation indices like the normalized difference red-edge index (NDREI) (De Luca et al., 2022; Grabska et al., 2020; Praticò et al., 2021; Solano et al., 2019).

Hence, this study uses two Copernicus Sentinel-1 and Sentinel-2 sensors for savanna rangeland assessment. These sensors operate in different ways and with different abilities. For instance, Sentinel-2 (optical) is most effective in clear sky conditions and produces highly precise vegetation results, though some of its components, such as the normalized difference vegetation index (NDVI), can become saturated in areas with dense vegetation. Sentinel-1 (synthetic aperture radar or SAR) operates under ecological conditions with more sensitivity to dielectric and land surface structures. Even though SAR remote sensing methods have shown their capability to acquire spatial data from vegetation, radar backscatter from polarimetric SAR techniques allows a comprehensive explanation of various vegetation parameters (Abdel-hamid et al., 2020). It has been reported that mesic/wet rangelands with high rainfall that causes high moisture content in vegetation and soil, fire is not commonly occurring in these rangelands (Sibanda et al., 2021). Yet there is still limited scientific evidence about the use of S1 and S2 data to monitor mesic/wet tropical rangelands after disturbance. Although, both optical and radar were stated to be sensitive in different ways to the various vegetation properties and background scattering or reflection processes produced by the sensor-specific characteristics (Urban et al., 2021).

Therefore, the primary aim of this study is to assess the impact of seasonal and fire occurrence on biophysical parameter distribution in mesic rangelands of Limpopo, South Africa, using Sentinel-1 and Sentinel-2. Specifically, to (1) determine the seasonal correlation between AGB and leaf area index (LAI) and the significant relationship among variables recorded by single spatial monitoring and, (2) to develop seasonal models for mapping and predicting the seasonal AGB in the study areas.

## Materials and methods

### Study areas

**Fig. 1:**
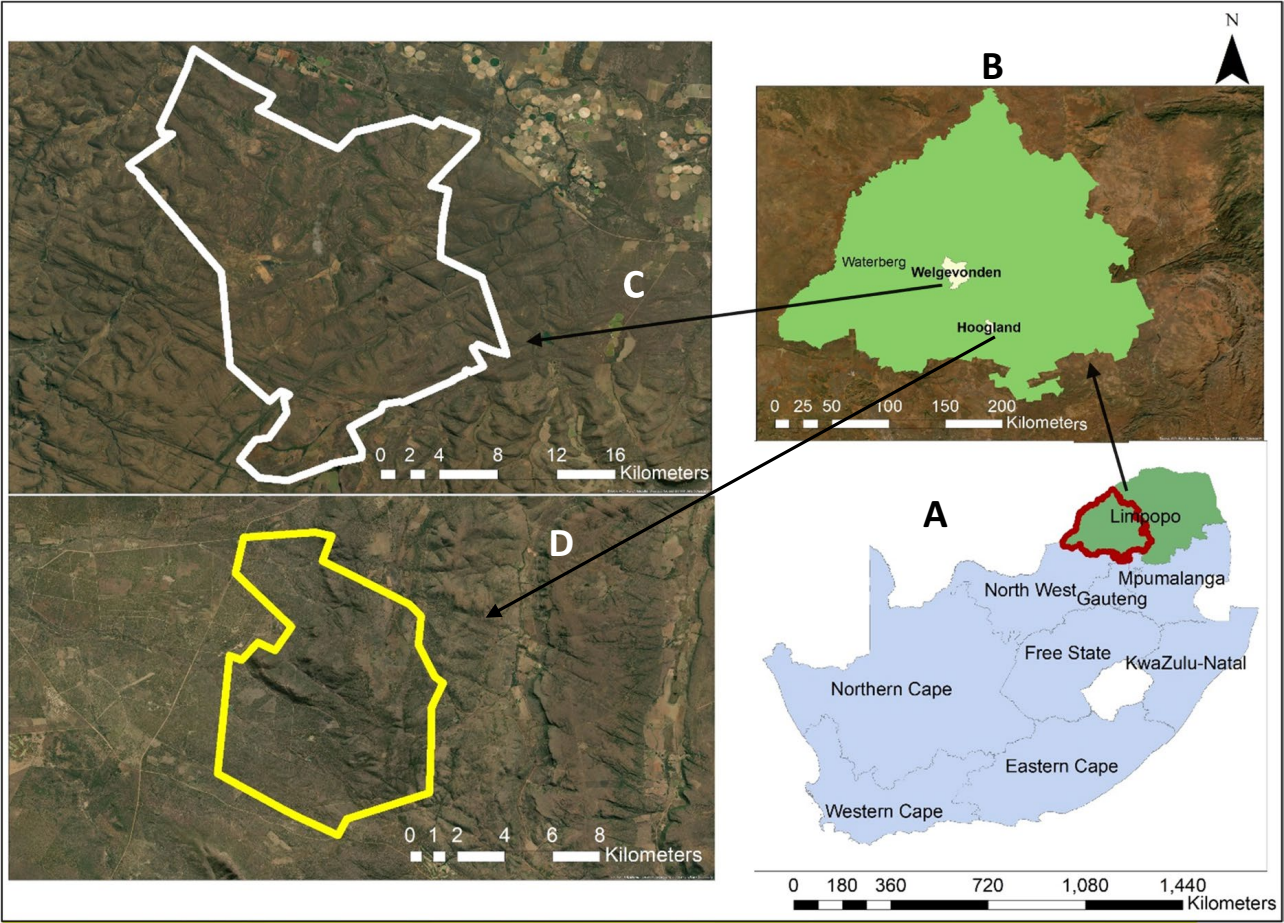
Illustration with two study sites in the Waterberg region of Limpopo province, South Africa, Welgevonden and Hoogland. **A** The map of South Africa with provinces, Limpopo province, highlighted in green. The red highlighted region represents the Waterberg district within the Limpopo province, **B** A close-up view of the Waterberg region, showcasing two specific study sites, **C** Welgevonden and **D** Hoogland game reserve

This study was conducted in two privately owned game reserves: Welgevonden (24°10′.24°25′S; 27°45′.27°56′E) and Hoogland (24°43′20.8′S; 28°07′48.7″E), in the Waterberg Estate, Limpopo Province, South Africa (see Fig. 1). The Waterberg lies north of the Bushveld Basin, where it forms a highland area. The highest part of the area is in the south Kransberg in the southwest towers out above the Limpopo Plain at the foot of the cliff-like escarpment made up of Waterberg Sandstone. The climate is predominantly warm to hot (mean minimum temperature is 14.4 °C, and mean maximum temperature is 44.9 °C) during summer and receives Mean Annual Precipitation (MAP) of 790–1174 mm, with mean annual evaporation between 1750 and 1900 mm (Institute of Soil, Climate & Water, 1999; Nesamvuni et al., 2003). Soil types are mainly red Hutton and Avalon form (Loxton, Venn and Associates, 1985). The vegetation type is primarily sour veld, which comprises Lowveld Sour Bushveld, patches of the North-Eastern Sourveld in the North, and South-Eastern Sourveld in the South, as described by (Acocks, 1988). The vegetation in this area is dominated by tropical savanna vegetation, which results to the Sourveld vegetation type. This area also comprises Lowveld Sour-Bushveld, which is found in the North-Eastern (Sourveld) areas of the North, and in the South-Eastern (Sourveld) areas of the South (Mbedzi et al., 2021; Nthakheni, 2006). Over the past three decades, the Welgevonden Game Reserve has been subject to natural management practices, ensuring the protection of its vegetation from external factors that could cause the complete removal of plant life, such as wildfires. Despite their typically low fire risk, the Hoogland Game Reserve experienced a severe wildfire in 2017.

### Sampling design and data collection

**Fig. 2:**
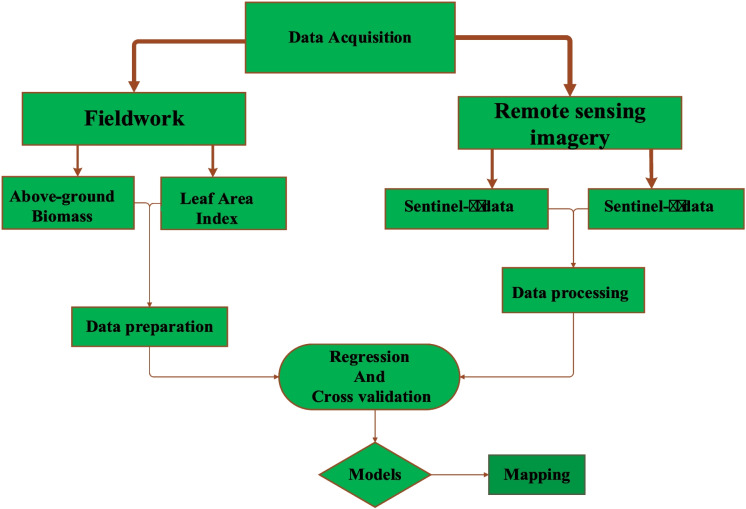
Flow chart of conceptualizing the procedures followed in this study

Six homogenous vegetation units (HVUs) were identified in the Hoogland and Welgevonden reserves through visual observations, i.e., three areas in each of the two reserves. A total of six yielded areas were randomly selected in both reserves with a range of vegetation cover and standing biomass. There is a distance of 500–900 m between each area. Within the areas, the transect was taken with a combination of systematic placement and a purposive sampling plot. The grasses were identified at species levels that belonged to different families and were grouped according to their families, such as the grass, grass-like, and non-grass species (forbs, sedges) along the 200-m transect. Then within the transact, each area was subdivided into 10 plots of 30 × 30m with homogeneous vegetation to capture variability. A total of 10 quadrats of 1 m^2^ were randomly located in each plot and then moved in each sampling time to avoid re-sampling. Each quadrant was sampled for different biophysical parameters. A total of 180 quadrats were sampled at the end of the experiment period. On each subplot, the information for main grass samples were collected for measurements of biophysical parameters during each season (Fig. 2). The vegetation parameters were measured and collected in November–December 2020 (for early-summer); July–August 2021 (winter), and March 2022 (late-summer). The grass samples were cut and oven-dried at 70 °C for 48 h. The grass leaf area index (LAI), vegetation canopy (VC), and the cover was measured directly from standing grass at the plot level.

#### Leaf area index

For accurate vegetation canopy analyses under each subplot, the seasonal leaf area index (LAI) was measured using a coptometer, following a widely-used accurate inter-row point method protocol, as described by Francone et al., (2014). The AccuPAR LP-80, ceptometer model manufactured by “Decagon Devices” (Pullman, USA) was used to evaluate the LAI of vegetation, determined from a leaf angle distribution variable measured from above and below the canopy. Ten readings were taken at each point for photosynthetic active radiation (PAR). This method is used to assess the gap portion according to a beginner’s version of sunny transmission model. The ceptometer uses fluctuations in PAR to calculate the LAI. Alongside the wand of the ceptometer, eighty 1 cm^2^ sensors calculate the PAR of the particular canopy, and these measurements are combined into a single value of LAI using the inversion technique (Palmer et al., 2017).

#### Aboveground biomass

For each quadrate, the standing and litter vegetation was measured every season at the same time. The standing vegetation within each quadrate was clipped at the theoretical height of 6–8 cm from the ground, placed in paper bags, dried in an oven at 700 °C for 72 h, and weighed. The collected grass samples were weighed fresh and dry and then converted to the aboveground biomass (AGB) to kg/m^2^. The dried aboveground biomass (kg/m^2^) was used for advanced analysis and is referred to as biomass here. Then the litter, including all dead material from leaves and stems in each quadrate was gathered, dried, and weighed. The data from the subsamples were then used to characterize the grassland growth status of the corresponding sample area.

### Remote sensing data and processing

The current study assumes that optical and synthetic aperture radar (SAR) data offer complementary remotely sensed data. The use of optical and SAR data in monitoring seasonal rangeland productivity allows for the detection of changes in rangeland productivity (vegetation cover, LAI, and biomass) throughout the growing season as highlighted by (Wang et al., 2019). Hence this study used these freely accessible data from ESA’s newly launched constellations of synthetic aperture radar (SAR) (Sentinel-1) and optical (Sentinel-2). Sentinel-1 is a C-band that offers backscatter coefficients at various polarizations. While Sentinel-2 is an optical sensor with an improved spectral resolution, with bands in the red-edge spectral parameters, with red-edge–based indices and traditional-based indices derived from S2 bands that may offer new chances for AGB assessment (Nuthammachot et al., 2022). Table 1 shows the bands from S1 and S2 that were used in this study.

**Table 1:** Sentinel-2 bands and Sentinel-1 features used in this study

Bands/C-bands	Description	Resolution
B2	Blue	10
B3	Green	10
B4	Red	10
B5	RedEdge1	20
B6	RedEdge2	20
B7	RedEdge3	20
B8	NIR	10
B8A	NNIR	20
B11	SWIR1	20
B12	SWIR2	20
VH	Vertical/horizontal	5 × 5
VV	Vertical/vertical	5 × 5

#### Sentinel-1-synthetic aperture radar

Sentinel-1 SAR (S1) image data cover up the study area was attained from Google Earth Engine (GEE) (https://code.earthengine.google.com). As S1 is part of the Copernicus program, it transmits a single C-band “synthetic aperture radar” tool functioning at the middle of 5.405 GHz frequency, and collects a constellation of two satellites (S1A and S1B) to guarantee data stability (Abdel-hamid et al., 2020). The S-1A and S-1B were set up between April 2014 and April 2016, offering a minimal 6-day repeat data series (12 days in South Africa), permitting constant vegetation assessment. S-1 functions in four image forms with numerous observation approaches, swath widths and spatial resolutions—the “Strip Map mode”, “Extra Wide swath mode”, “Interferometric Wide swath mode”, and “Wave swath mode”. A total of 24 scenes of S-1 data were attained, covering three growing seasons from 2019 to 2022. All the attained data were from dual polarization VV/VH, obtained in the “Interferometric Wide swath mode” in the ground range noticed structure. They were in a variety and high resolution of 5–20 m (Abdel-Hamid et al., 2020). The VH and VV backscatter has been commonly used for biomass estimation, but it is highly affected by topography. The intensity ratio variables are less affected by landscape than intensities, but they show significant changes in bare surface areas due to soil moisture/roughness. This study developed a time series analysis using dual polarization VV and VH data of the 2019, 2020, 2021, and 2022 growing seasons.

#### Sentinel-2-optical

The S2 constellation comprises two orbiters, namely S2A (launched in November 2015) and S2B (launched in August 2017). S2B is currently operational, and the revisit time is approximately 5 days. This study utilized data obtained between 2020 and 2022 from the optical S2 constellation using the Google Earth Engine (GEE) (https://code.earthengine.google.com). [[Each of the two orbiters (S2A and S2B) / In addition to the two orbiters, the constellation?]] contains 13 bands that cover the spectrum from visible to short wave infrared (SWIR) with a high spatial resolution of 10 meters (Urban et al., 2021). The images were seasonally captured at approximately 10:30 am local time. Sixteen vegetation indices (VIs) were developed for biomass (AGB) assessment (Table 2). For mapping the AGB of rangelands using S2, indices such as NDVI were described as very appropriate, since they give 10 m spatial resolution, and most commercial software supports NDVI analysis with standardization (Malhi et al., 2022).

**Table 2:** Vegetation indices used

Index	Equation	Bands	References
“NDVI (normalized difference vegetation index)	*(nir − red)/(nir + red)*	Red, nir	Rouse et al., 1974
NRVI (normalized ratio vegetation index)	*(red/nir − 1)/(red/nir + 1)*	Red, nir	Baret & Guyot, 1991
WDVI (weighted difference vegetation index)	nir − s * red	Red, nir	Richardson & Wiegand, 1977
RVI (ratio vegetation index)	*red/nir*	Red, nir	Gorai et al., 2014
TTVI (Thiam’s transformed vegetation index)	*sqrt(abs((nir − red)/(nir + red) + 0.5))*	Red, nir	Thiam, 1997
TVI (transformed vegetation index)	*sqrt((nir − red)/(nir + red) + 0.5)*	Red, nir	Deering et al., 1975
KNDVI (kernel normalised difference vegetation index)	*tanh(((nir − red)/(nir + red)))^2*	Red, nir	Camps-Valls et al., 2021
MSAVI (modified soil adjusted vegetation index)	nir + 0.5 − (0.5 * sqrt((2 * nir + 1)^2 − 8 * (nir − (2 * red))))	Red, nir	Qi et al., 1994a
MSAVI2 (modified soil adjusted vegetation index 2)	(2 * (nir + 1) − sqrt((2 * nir + 1)^2 − 8 * (nir − red)))/2	Red, nir	Qi et al., 1994b
CTVI (corrected transformed vegetation index)	*(NDVI + 0.5)/sqrt(abs(NDVI + 0.5))*	Red, nir	Perry Jr & Lautenschlager, 1984
NDREI1 (normalised difference red edge index 1)	*(redEdge2 −redEdge1)/(redEdge2 + redEdge1)*	Rededge2, rededge1	Mauya & Madundo, 2021
NDREI2 (normalised difference red edge index 2)	*(redEdge3 − redEdge1)/(redEdge3 + redEdge1)*	Rededge3, rededge1	Mauya & Madundo, 2021
MCARI (modified chlorophyll absorption ratio index)	((redEdge1 − red) − (redEdge1 − green)) * (redEdge1/red)	Green, red, rededge1	Daughtry et al., 2000
CLRE (red-edge-band chlorophyll index)	*redEdge3/redEdge1 − 1*	Rededge3, rededge1	Mauya & Madundo, 2021
SATVI (soil adjusted total vegetation index)	(swir2 − red)/(swir2 + red + L) * (1 + L) − (swir3/2)	Red, swir2, swir3	Marsett et al., 2006
SLAVI (specific leaf area vegetation index)	(nir − red) * (1 + L)/(nir + red + L)	Red, nir	Lymburger et al., 2000

### Climate data

The study obtained climatic data from the Google Climate Earth Engine website (https://climateengine.com) for the Waterberg region in Limpopo, South Africa. The data comprised daily records of precipitation and temperature for the period from 2019 to 2022, which was then converted into monthly averages. The study area was defined using a polygon covering the Hoogland and Welgevonden areas of the Waterberg region. The study utilized precipitation data derived from the “Climate Hazards Group Infrared Precipitation with Station” (CHIRPS) records. The CHIRPS dataset provides daily rainfall data for a quasi-global coverage of 50°N–50°S since 1981, with the most recent version, Version 2.0, belonging to the satellite-gauge category (Abdel-Hamid et al., 2020). These sources of data and methods of processing are crucial for accurate and reliable climatic data analysis, which is necessary for informing environmental and natural resource management decisions.

### Data analysis

#### Correlation analysis

Pearson correlation is a statistical procedure used to determine the strength and direction of the linear relationship among two continuous parameters. In this case, it was used to establish the correlation between aboveground biomass (AGB) and remote-sensing derived variables. It is important to consider the correlation coefficient’s magnitude and significance. A correlation coefficient close to −1 or 1 shows a strong linear correlation among the variables, while a coefficient close to 0 indicates a weak or no linear relationship. The significance level indicates the chance that the identified correlation coefficient could have occurred by chance, and a significance level of less than 0.05 (*p* < 0.05) is typically considered statistically significant (Benesty et al., 2009).

#### Model development for AGB prediction

Multiple linear regression analysis was done with cross-validation to select the individual parameter with the highest coefficient, and those associated significantly (i.e., *p* value < 0.05) with the AGB were chosen (Adame-Campos et al., 2019). Then to establish individual models for AGB estimation bands (S1 and S2), different vegetation indices, LAI and their combination were used. Random forest algorithm (RF) was used to develop AGB seasonal models from LAI and remotely sensed data. Random forest works by creating multiple decision vegetation from random subsets of the training data. Each decision vegetation is constructed by selecting a random subset of the features and using them to “recursively split” the data into smaller subsets created on the values of those elements. This process continues until each subset contains only a single class or until some stopping criteria are met. The final prediction of the model is made by taking the mode (in the case of classification) or the mean (in the case of regression) of the predictions of all the individual vegetation (Table 3).

**Table 3:** Model scenarios were developed using the leaf area index, bands and different indices (from Sentinel-1 and Sentinel-2)

Model	Possibilities
1	LAI + Bands+ Rededge + Traditional indices
2	LAI + Bands+ Rededge
3	LAI + Rededge + Traditional indices
4	LAI + Bands+ Traditional indices
5	Bands + Rededge + Traditional indices
6	Rededge + Traditional indices
7	Bands+ Rededge indices
8	Bands+ Traditional indices
9	LAI + Traditional indices
10	LAI + Bands
11	LAI + Rededge
12	Red-edge
13	LAI
14	Bands
15	Traditional
16	LAI + Bands+ Rededge + Traditional indices + VH + VV
17	LAI + Bands+ Rededge + VH +VV
18	LAI + Bands+ VH + VV
19	LAI + Traditional indices + VH + VV
20	LAI + VH + VV

Three significant steps were followed in the modeling procedures that include (1) variable selection, (2) model development/fitting, and (3) validation of the model as highlighted by Mauya & Madundo, (2021) (caret package and VSURF package implemented in Rx64 3.4.0 software). At the same time, stepwise multiple linear regression (SMLR) was also used during model development. SMLR is a statistical technique used to build a linear regression model by selecting the most significant predictors from a set of independent variables. SMLR is an iterative process that involves adding and removing predictors from the model, one at a time, until the best model is obtained (Silva et al., 2017).

To assess the accuracy of the model, several statistical measures, including the coefficient of determination (*R*^2^), root mean square error of prediction (RMSE), mean absolute error (MAE), and relative RMSE, were used. These metrics were calculated by dividing the RMSE by the measured mean and multiplying by 100%. This approach was adopted based on the statistics precision system of measurements and the work of Ramoelo & Cho, (2018).

Furthermore, an analysis of variance (ANOVA) statistical method was employed to evaluate the significant differences in the seasonal dynamics and fire impact on the aboveground biomass (AGB) distribution of tropical rangelands. This method is a common statistical approach used to assess the variation among groups and is useful in identifying significant differences between variables.

## Results

### Correlations between individual predictor variables of aboveground biomass

Table 4 shows Pearson’s correlation analysis between all spectral variables, leaf area index and AGB, and their significant correlation throughout the season. The correlation coefficients were determined as quantitative indicators of the compliance of the AGB for each season. The correlation coefficients for all evaluated indicator variables range from 0.001 to 0.111. Overall, the correlations were poor, but they varied among each other and season. Despite the poor correlations, the relationships were statistically significant (*p* < 0.05) with AGB. The indices SLAVI and NDREI1 and the spectral bands red-edge (B6 and B7) and NIR (B8 and B8A) correlated most strongly with AGB during early summer (*p* < 0.05). During winter, MCARI and B2 were the most significant variables (*p* < 0.001) with LAI (*R*^2^ = 0.052), while NDREI2 (*R*^2^ = 0.042) proved important variables during later summer.

**Table 4:** Summary of the aboveground biomass (AGB) estimation performance using LAI, bands, and various vegetation indices (red-edge and traditional-based) as predictor variables

Seasons	Variables	*R*^2^	RMSE (kg/ha)	RRMSE%	*P*<0.05
	WDVI	0.057	0.14	46.476	0.035*
	NDREI1	0.060	0.14	46.391	0.028*
	NDREI2	0.055	0.14	46.509	0.036*
Early-summer	SLAVI	0.093	0.13	45.58	0.006**
	CLRE	0.056	014	46.501	0.035*
	B2	0.069	0.13	46.164	0.0184*
	B6	0.102	0.13	45.334	0.003**
	B7	0.109	0.13	45.164	0.003**
	B8	0.085	0.13	45.777	0.009**
	B8A	0.100	0.13	45.391	0.004**
	NDREI1	0.111	0.13	45.119	0.003**
Winter	MCARI	0.064	0.13	46.292	0.023*
	B2	0.051	0.14	46.618	0.044*
Later-summer	LAI	0.052	0.10	25.310	0.042*
	NDREI2	0.042	0.010	29.461	0.037*

*Significance level of 0.05 and **significance level of 0.01

Based on the cross-validation results, 20 models were obtained using two modeling algorithms, namely RF and SMLR, with the spectral variables. The fitting results of the better models are summarized in Table 5. The values of *R*^2^ in parametric SMLR were slightly lower than those obtained from the nonparametric method (RF). The values for RMSE, RRMSE, and MAE acquired using the nonparametric method (RF) were high throughout the season. The random forest models were identified as the best in all seasons based on the metrics values (*R*^2^ ranges from 0.326 to 0.517 and RMSE = 0.069 to 0.335). The most important variables for the various predictors in the models are presented in Table 6. These results show that RF models, considered the best model, had higher accuracies of AGB estimation than SMLR models. The best models were then used to plot and map the seasonal distribution of AGB.

**Table 5:** Best models derived from stepwise multiple linear regression (SMLR) and random forest regression (RF)

Season	Modeling algorithms	Model	*R*^2^	RMSE	RRMSE	MAE
Early-summer	SMLR	3	0.335	0.122	37.608	0.010
Early-summer	RF	16	0.437	0.110	25.233	0.090
Winter	SMLR	17	0.314	0.123	39.438	0.102
Winter	RF	3	0.326	0.120	21.070	0.010
Later-Summer	SMLR	4	0.246	0.088	24.306	0.065
Later-Summer	RF	13	0.517	0.069	10.68	0.052

**Table 6:** Importance variables from the best model

Season	Model	Selected
SMLR (early-summer)	3	B2; B4; B7; B8; B8A;B12;KNDVI;MSAVI;MSAVI2
RF (early-summer)	16	SLAVI + NDREI1; B3
SMLR (winter)	17	B5;B6;B7;B11;B12;VV
RF (winter)	3	LAI;B11;B4
SMLR (later-summer)	4	LAI; RVI; NDREI2; NDREI2
RF (later-summer)	13	LAI;NDREI2

**Fig. 3:**
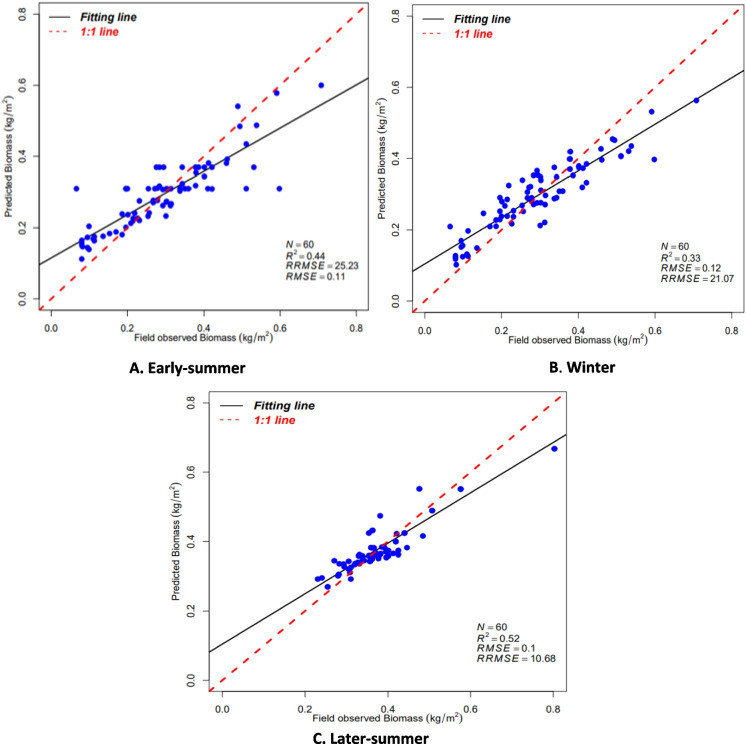
The scatterplots of the predicted and estimated AGB from different RF models throughout the season

Scatterplots show the relations between the predicted values and observed data and can describe the prediction performance (Li et al., 2022). In this study, the associations between predicted AGB from different models throughout the season against observed AGB are shown in Fig. 3. The best AGB estimation models were observed from each season’s RF algorithm. They were considered better suited for AGB prediction throughout the season due to their high *R*^2^ and lower RMSE and RRMSE, since these metrics were used to differentiate between the models.

**Fig. 4:**
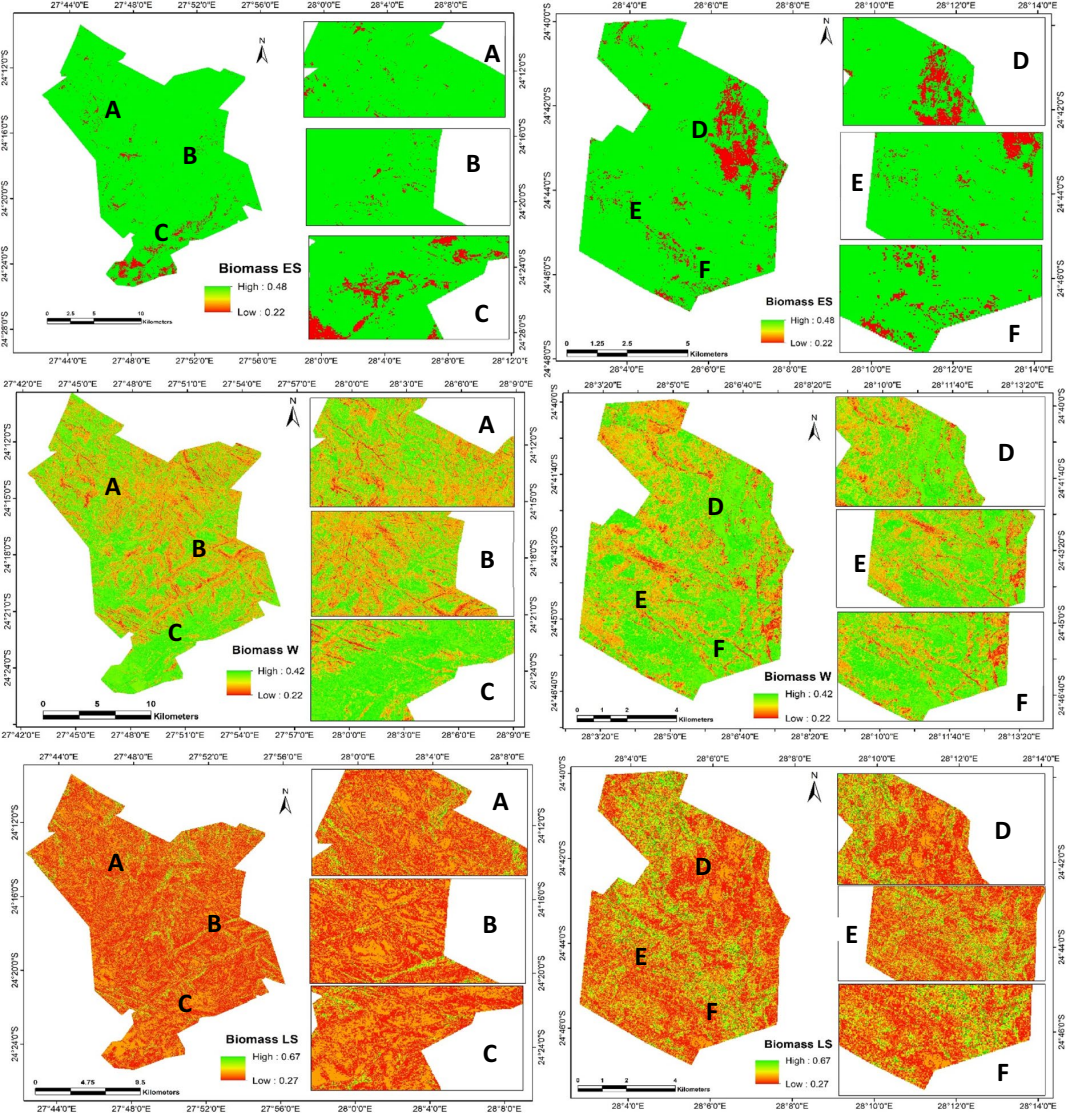
Spatial distribution of seasonal AGB (kg/m^2^) derived from RF models. 1 Spatial distribution of AGB during early-summer (kg/m^2^), 2 Spatial distribution of AGB during winter ((kg/m^2^), 3 Spatial distribution of AGB during late-summer (kg/m2). Selected or sample areas are marked with A, B, C, D, E, and F, respectively

These inputs from the RF models were used to produce three different AGB maps for the seasonal evaluation in this study, as indicated by the realistic seasonal fluctuation of AGB in Fig. 4. These results indicate that the particular distribution and variation of AGB is affected by several factors, including season. This study shows a variation in the seasonal distribution with the highest AGB (0.42–0.47) and lowest (0.21–0.27) throughout the season. The variability in AGB is most evident during periods of peak productivity, with the highest levels occurring in late summer, followed by early summer, and decreasing during winter (the cold–dry season). Early summer and winter show no significant difference in the spatial distribution of the AGB. Moreover, no significant difference in the distribution of AGB was exhibited throughout the season. There was an interesting distribution of AGB during the later summer, where Hoogland showed a balanced distribution of AGB, which was associated with the fire that occurred in that game reserve in 2017. The analysis of variance (ANOVA) showed that fire significantly affects the seasonal distribution of AGB. The area with fire occurrence (Hoogland) (ranging from 0.26 to 0.39 kg/m^2^) shows significantly higher AGB than the non-fire area (Welgevonden) (0.24–0.35 kg/m^2^) throughout the season (Table 7).

**Table 7:** The impact of fire on the seasonal distribution of AGB in mesic rangelands

	Early-summer			Winter			Later-summer		
Area	Mean (kg/m^2^)	Std	CV (%)	Mean (kg/m^2^)	Std	CV (%)	Mean (kg/m^2^)	Std	CV (%)
Welgevonden	0.24	0.13	55.45	0.29	0.09	34.83	0.35	0.06	15.70
Hoogland	0.37	0.13	35.02	0.26	0.01	38.23	0.39	0.11	27.5
SL	*	NS	**	*	NS	NS	*	*	**

*SL* Significant level. Significant at *p ≤ 0.05 **p≤0.01, but NS is not significant at *p*≥0.05. Standard deviation (Std) and coefficient of variation (CV)

Likewise, Sentinel-1 was eliminated in our models during AGB estimation throughout the season. Therefore, the study tested the evolution of Sentinel-1 backscatter to understand the relationship between AGB and Sentinel-1 variables. The trend of radar features is usually evaluated to observe the temporal changes of vegetation in particularly stressed rangelands (either too dry or wet). Most studies use SAR data during the vegetation assessment, focusing on the backscattering intensity and polarimetric variables of individual sections and trends (Urban et al., 2021; Santoro et al., 2015; Schuster et al., 2011).

**Fig. 5:**
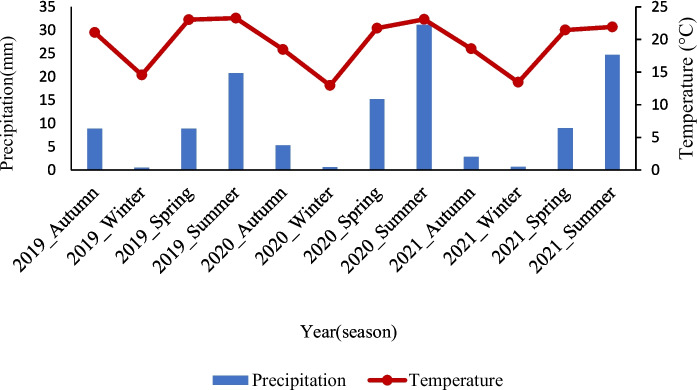
Overview of the seasonal mean temperature and rainfall in the Waterberg region (2019–2021)

Due to climate change, this study also acquires the mean temperature and precipitation throughout the season from 2019 to 2021 in the Waterberg region from the climate earth engine (see Fig. 5). These results cover both study areas (Welgevonden and Hoogland). Since they are in the same region, this adopted them to explain the ecological condition before and after the study period. The results highlighted the seasonal changes between the two ecological parameters of the Waterberg region. The region receives an amount of annual rainfall that ranges between 0.50 and 35 mm, where the highest rainfall occurs between spring and summer, and the least in winter. This rainfall is accompanied by increased mean yearly temperature, with higher mean temperature in the hot–wet summer season (Fig. 5).

**Fig. 6:**
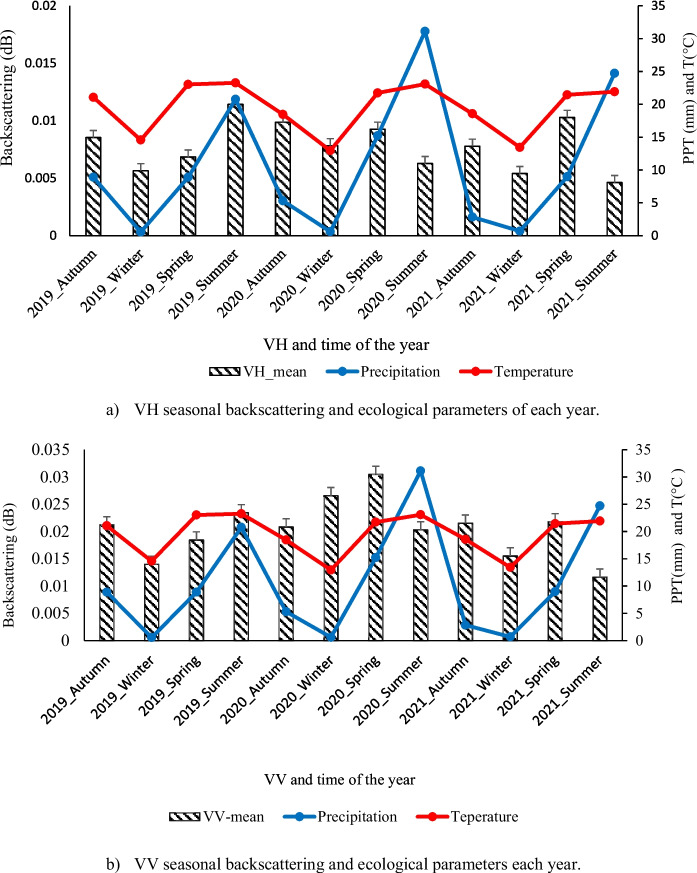
Comparing seasonal SAR backscattering and ecological parameters over rangeland of the Waterberg region, **a**–**b** VH and VV backscattering during the 2019–2021 growing seasons, the x-axis of the figures indicates the season of the respective years

The evolution of VV and VH polarization was acquired throughout the season in the Waterberg region. VH and VV polarization signals strongly correlate C-band with ecological parameters in all seasons because of the high sensitivity to vegetation AGB. Figure 6 shows the seasonal backscattering VH and VV polarizations in savanna rangelands in the study area. The signals of AGB in VH backscattering change with the season as the temperature and rainfall increase; this leads to a change in vegetation structure. Although very high rainfall and temperature can lead to low dB values, as occurred in these results during the 2020 summer season. Balanced ecological parameters, like in the 2019 summer, produce a stable dB production.

The decrease was noted with the decline in ecological parameters such as temperature and rainfall during the winter. The same decrease was perceived in VV sequential in this study. The extensive range noted in this study can be ascribed to the scattering caused by the grass leaves structure during this season. The season’s signal variation, particularly in VH, is derived from two processes that dominate during the growth cycle of grasses: double-bounce scattering and volume scattering (Abdel-hamid et al., 2020). The highest dB values in polarizations were associated with the period in which the AGB generally reaches its maximum.

**Fig. 7:**
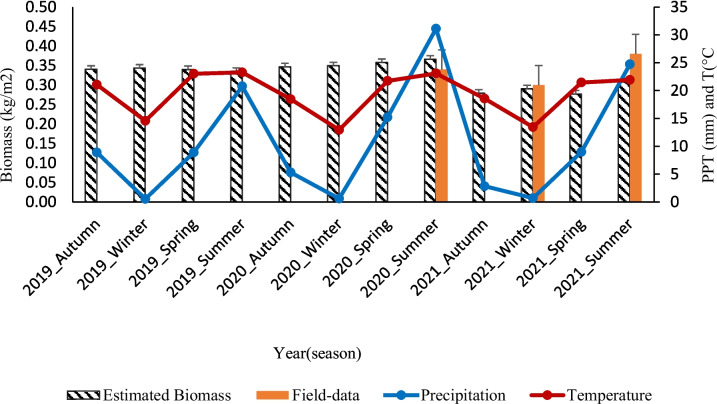
The seasonal trend of estimated and field AGB (kg/m^2^) overlaid on mean temperature and rainfall (2019–2021); the x-axis of the figures indicates the season of the respective years

Figure 7 illustrates the correlations between the seasonal SAR mean of estimated AGB, field AGB, and ecological parameters over the rangeland. Results revealed no significant difference in estimated AGB between the 2019 and 2020 growing seasons. These results were associated with the same range of ecological parameters (rainfall and temperature) during this time. During the 2020 summer season, high rainfall and average temperature occurrence led to a drop in AGB in all seasons. This decline was also proved by field AGB data from the 2020 to 2021 period, although the 2021 summer shows some productivity improvement with less rainfall compared to the 2020 summer (rainy) season.

## Discussion

This study observed the influence of ecological factors, namely climate (seasonal variation) and rangeland disturbance, on aboveground biomass (AGB), which are considered key factors in rangeland conditions by Sankaran et al., (2005). Additionally, the selection of independent variables was significant for remote sensing based AGB prediction models, and possible parameters from the images, such as individual bands, vegetation indices, and the field LAI, were used due to their correlation with rangeland AGB. In this regard, the analysis of Pearson correlation coefficients of individual spectral parameters, LAI, and the AGB of different seasons, indicates that only 13 spectral variables such as SLAVI, NDREI1 indices, red-edge (B6 and B7), NIR bands (B8 and B8A), and LAI significantly correlate with AGB simultaneously (Calders et al., 2020). Most spectral variables occurred in early summer when the vegetation was still approaching its peak production (at the flowering stage), and most variables fully reflect vegetation characteristics, especially AGB. These results suggest that remote sensing data do not fully reflect individual features in AGB assessment during different seasons (Lu, 2006; Zheng et al., 2004). The results in this study are also in agreement with the observation of Nguy-Robertson et al., (2012), who noticed that the spectral bands with red and near-infrared bands and vegetation indices, namely traditional (SLAVI and WDVI) and red-edge indices (NDREI1 and NDREI2), lead to better AGB estimation, especially during the vegetation stage, and they are perfect at the species level.

In this regard, the high activity of red-edge spectral variables was associated with the fact that during the flowering stage, plants experience an increase in chlorophyll content that enhances the plant’s ability to absorb light. This leads to an improvement in the red-edge indices as the red-edge is directly related to chlorophyll content. This stage is also known for its high photosynthetic activities and high light absorption, leading to an increase in plant growth and biomass. Furthermore, the increased photosynthetic activity results in improved red-edge indices, as the red-edge is usually directly related to photosynthetic activity. Although they are less reflected during peak productivity, this study used the LAI as the best alternative for AGB estimation during peak season, as highlighted (Aklilu Tesfaye & Gessesse Awoke, 2021; Gu et al., 2013; Xie et al., 2018). Likewise, it has been reported that the point of maximum production on the bands and indices with red-edge are more sensitive to AGB variation in green vegetation, unlike ageing vegetation, and therefore less susceptible to saturation problems (Mutanga & Skidmore, 2004). These observations were similar to the findings of Mutanga et al., (2012), Chen et al., (2009), and Mutanga & Skidmore, (2004), who noticed poor correlations between the AGB and standard NDVI’s, and associated these results with the reflection of the saturation level reached on thick flora.

This study observed the best predictive models from the RF modeling algorithm, which was used to plot and map the seasonal distribution of AGB. The best models in this study included the texture of NDREI2 derived from the red-edge components. It has been widely stated that the red-edge components are essential in AGB modeling because they are more sensitive to moisture and shade mechanisms characteristic of vegetation, such as savanna land with a mixture of shrubs and forbs (stand structure) (Mauya & Madundo, 2021). They also result in atmospheric conditions having less influence on spectral signatures, thus reducing noise in the models (Gao et al., 2018, 2011; Mauya & Madundo, 2021).

The red-edge components were identified as the most important variables for monitoring vegetation changes over time, as they provide valuable information for understanding the impact of ecological factors, such as land use change and climate change. In this study, the most notable dominant of the red-edge components was during the later summer when the grasses were in the booting/heading stage (peak production). The leaves are fully mature at this stage, and photosynthetic processes are less active. These results were similar to the observations of Kanke et al., (2016), Zhao et al., (2016), Peng & Gitelson, (2012), and Ramoelo et al., (2012), who found that the red-edge indices had better sensitivity at low to medium chlorophyll levels. These results were associated with the advantages of red-edge indices, such as higher sensitivity in detecting the physiological state of plants at tall plant biomass or cover. This is associated with its location among the bands where strong light assimilation by plant pigments and high leaf reflectance occur. Further motivation for using red-edge based in the AGB assessment in this study was based on the mechanical structure and the position of indices between bands, where strong light absorption occurs (Kanke et al., 2016). The results of this study also confirm the theoretical claim mentioned above, as LAI was the most important predictor variable for AGB in late summer, which was considered the booting stage of vegetation (Chung et al., 2009; Forkuor et al., 2020; Kumar et al., 2020).

The LAI in this study was also considered as a powerful alternative to predict AGB, particularly during the later summer season, which is regarded as the peak productivity season of rangeland vegetation. During this period, grasses mature, and their leaves and stems reach maximum productivity (Kira et al., 2016). Hence, LAI was identified as a good predictor of biomass during peak production, as the relationship between leaf area and biomass production is well established for this time of the year. This results in a larger leaf area that generally corresponds to a larger photosynthetic capacity and, as a result, a higher potential for biomass production. The LAI is positively correlated with biomass, as the more leaves a plant has, the more photosynthetically active tissue it has, and the greater the potential for growth and biomass accumulation (Costanza et al., 2015).

Although it has been reported that the relationship between LAI and biomass is not always linear, the relationship between leaf area and biomass production is influenced by factors such as leaf age, nutrient availability, and species. This study’s results agree with other studies showing that LAI is a good predictor of AGB. For example, Vyvlečka & Pechanec, (2023), found a strong relationship between LAI and AGB in temperate deciduous forests. Similarly, Gao et al., (2011) found a strong correlation between LAI and AGB in a temperate grassland. Furthermore, Wang et al., (2019) observed a strong correlation between LAI and AGB in a subtropical grassland.

Overall, this study shows the AGB models’ predictive accuracy across seasons varied depending on the selected predictors during variable selection. This study also agrees with the observed and calculated AGB range for all seasons. RF models outperformed and accurately captured the seasonal variation in AGB in our study areas, where later summer shows better AGB values compared to other seasons. These observations were associated with vegetation structure, vegetation stage of vegetation, and ecological parameters, including temperature and precipitation. At this time, the vegetation is at the booting stage, where photosynthetic processes are slightly active as the foliage reaches its peak production. This could be due to the ecological parameters, such as the impact of high rainfall during this season. Most studies observed high AGB during this season, and some studies referred to this season as the end of the rainy season, and the vegetation has reached its maximum production stage. The foliage during this time is fully mature and is dominated by mature leaves, and the output is mainly dependent on the ecological parameters of the area (rainfall and temperatures) (Kaiser et al., 2012; Swemmer et al., 2007; Zhang et al., 2020).

On the other hand, rangeland disturbance in this study has a significant impact on mesic rangeland productivity, and as the results displayed a slightly higher AGB in Hoogland during late summer. The high AGB in Hoogland was associated with the occurrence of fires in this reserve in 2017; this stimulated the amount of vegetation with an increase in species diversity. This is also consistent with the observations of Grime, (2006), Wright et al., (2005), Trollope, (2011) and Trollope et al., (2002), who observed a shift in vegetation in tropical grasslands following disturbance by fire and human intervention. The changes or shifts in the rangeland vegetation also indicate the fluctuation in phenotypes of individual traits, which are a clear indication of rangeland productivity throughout the season. Therefore, it is easy to observe how the rate of evolution in rangeland parameters changes from physiological approaches associated with slow growth in seasons of low productivity to rapid growth in more productive seasons (Grime, 2006). The changes in mesic tropical rangelands can be predicted by measuring seasonal variation in AGB distribution and long-term sustainability in tropical rangelands using specific data from S1 and S2. S1 and S2 data are sensitive to various vegetation parameters, including the surface scattering mechanisms as observed by Urban et al., (2021).

However, the Sentinel-1 variables were eliminated during model development in this study. This elimination was due to S1’s sensitivity to the phenology-changing aspects of dense vegetation, where leaves and upper canopy characteristics take over the backscatter signals in the C-band (Wang et al., 2019). Further motivation for the elimination of Sentinel-1 variables is the high correlation with Sentinel-2 variables, which leads to multicollinearity in the model. This can result in unstable and unreliable model estimates and may decrease the significance of individual variables. As such, variables with high collinearity are generally eliminated from the model (De Beurs et al., 2005). Additionally, Sentinel-1 data may also show higher non-stationary patterns in space and time than Sentinel-2 variables, which can affect the accuracy of the biomass model. Therefore, variables with non-stationary patterns may be eliminated from the model, and alternative variables that are more stationary may be selected, as noted by Petropoulos et al., (2016).

This study examined Sentinel-1 backscatter data to assess the seasonality and occurrence of disturbances in mesic tropical rangelands. Two ecological parameters (temperature and precipitation) were diagnosed with S1 backscatter to identify the threat of climate change and management intervention in mesic tropical rangelands. The results showed that the evidence of seasonal variation in AGB was stronger in the VV backscatter than in the VH backscatter. These results agreed with some observations that used SAR data for rangeland monitoring and focused on analyzing SAR backscatter and biophysical parameters (such as AGB). For example, the study by Abdel-Hamid et al., (2020) and Voormansik et al., (2020) observed high backscatter in VV under the selected polarization during grassland assessment. Both polarizations, VV and VH, showed seasonal dynamics in tropical rangelands and provided insights to clarify the response of rangeland vegetation to variations in ecological parameters, precipitation, and temperature. Several studies observed high VH and VV AGB backscatter levels during the rainy season, with precipitation considered the main factor. The vegetation after high precipitation can lead to the high moisture content in vegetation and soil that significantly changes the SAR backscattering of grassland (Abdel-Hamid et al., 2020; Paloscia et al., 1999; Voormansik et al., 2020; Wang et al., 2019). The current study agrees with the aforementioned studies and associates the low signal of backscattering between the 2020 and 2021 summer with the sensitivity of SAR data to the water content on the observed surface.

Similarly, the sensitivity of SAR backscattering to vegetation structural changes starts from the early stage to booting stage (peak production), where water content is high in stem elongation and declines after peak production, as observed by Beriaux et al., (2021). In addition to high water content, this study also associates the decline of backscattering with the occurrence of fire in one of the study areas. Therefore, the response of SAR backscattering to fire disturbance also causes a significant signal decline that illustrates the effectiveness of dual-polarized data. This observation is primarily attributed to vegetation burning, either partially or entirely, leading to a reduction in volumetric scattering influence (Imperatore et al., 2017).

Overall, there was no significant difference between estimated AGB with SAR and field-measured AGB. Lower AGB values were observed in estimated data with SAR and field-measuring between the 2020 summer and 2021 summer growing seasons, where poor AGB production correlates with high rainfall in the 2020 summer. Since it is not fully known about the use potential to track the seasonal changes of mesic tropical areas after fire occurrence. This study found that the S1 backscatter data (SAR) can predict seasonal changes and disturbances in mesic tropical rangelands. It has been stated to be always appropriate to measure any vegetation type under ecological conditions (Mathieu et al., 2019; Wang et al., 2019).

## Conclusion

Remote sensing tools have been proven very efficient, particularly with the advent of easily accessible and open-source high resolution data and software. This study presented the results from the assessment of the impact of seasonal and fire occurrence on biophysical parameters distribution in mesic tropical rangelands of Limpopo, South Africa, using S-1 and S-2 data. The field data and spatial AGB were used for training and cross-validation in SMLR and RF algorithms. The individual correlation between AGB and LAI and single spatial parameters were tested, and recorded the significant relationship among the variables.

The best models in this study were retrieved from RF throughout the seasons. A seasonal variation of AGB in mesic tropical rangelands was related to fire occurrence in one of the study areas. Ecological parameters, particularly rainfall, mainly influenced the correlation between VH/VV backscattering. Obtaining maps of AGB with a good level of accuracy achieved is a significant advantage in improving further observation and monitoring of the mesic tropical rangelands and carrying out an effective and more targeted management intervention. The results obtained in this work will be necessary for developing intensive monitoring on the integrated use of SAR and optical data for monitoring the study area over time to assess the mesic tropical rangeland response to fires.

The findings of this study contribute to poverty mitigation by improving natural resource management interventions and addressing the goals for environmental protection with ongoing climate change occurrence (2021); IPBES (2019); Intergovernmental Panel on Climate Change (IPCC) (2011) in rangelands. These efforts align with Sustainable Development Goal 15 (SDG 15), which emphasizes life on land and its role in sustaining food security and improving the well-being of humankind at various levels, from local to global. However, upcoming studies could validate AGB assessments over extended periods of burning at larger or international geographic areas, such as dry tropical rangeland and forestry regions with substantial biomass. Furthermore, stakeholders for carbon trade policies and forest management approaches can benefit from AGB assessments based on the proposed practice.

## Data Availability

All the Sentinel data are free of cost and are in the open domain, and field data port the published claims and fulfill with field requirements.
